# Inadequate food safety knowledge and hygiene practices among street food vendors in Dhaka, Bangladesh

**DOI:** 10.1038/s41598-024-68099-y

**Published:** 2024-07-29

**Authors:** Sufia Islam, Nafisa Tanjia, Amal K. Mitra, Afjal Hossain, Mahajuba Tanija Jasika, Suhana Sara Suhi, Sheikh Jamal Hossain

**Affiliations:** 1 Department of Pharmacy, East West University, Aftabnagar, Dhaka Bangladesh; 2 Department of Public Health, Julia Jones Matthews, School of Population and Public Health, Texas Tech University Health Sciences Center, Abilene, TX USA; 3 Global Health and Migration Unit, Department of Women’s and Children’s Health, Uppsala University, Uppsala, Sweden; 4 Maternal and Child Health Division (MCHD), Icddr,b, Mohakhali, Dhaka 1212 Bangladesh

**Keywords:** Food safety, Hand washing, Knowledge, Food-borne illness, Vendors, Diseases, Health care

## Abstract

Food safety remains a critical issue with outbreaks of foodborne illness. The knowledge gap of food safety and improper hygienic practices of food handlers are the key factors for the transmission of foodborne diseases. This study was conducted to investigate the level of food safety knowledge and practices among street food vendors in Dhaka City, Bangladesh, and its implications on consumers’ health. This cross-sectional study was conducted among 350 respondents in seven areas of Dhaka City. Trained data collectors gather data by interviewing street vendors using a pretested questionnaire. Most of the vendors (98%) were male, with 48% having secondary education and 85% having no food safety training. Although about 89% of the vendors were found to practice hand washing, only a small proportion of them practised using hand gloves while handling raw products (5.6%), cleaning tables (2.2%), preparing foods (1.3%) or handling garbage (0.9%). The education level of the vendors and their work experience were significantly correlated with their hand washing practice, wearing hand gloves and their knowledge about food-borne illnesses. The study demonstrated that formal education played a significant role in vendors’ knowledge and practice of health safety measures for food handlers to prevent foodborne illness. Effective food safety training and monitoring are needed to increase vendors' knowledge and practices, and in reducing foodborne diseases.

## Introduction

The street food sector in developing countries has experienced significant growth due to rapid urbanization and evolving consumer habits. However, there are persistent concerns about health risks stemming from unregulated practices^1^. Street food vending is widespread in developing countries, particularly in Asia, Africa and Latin America. It is part of an informal food supply sector characterized by largely unregulated practices^2^. Maintaining good personal hygiene and food handling practices is crucial for preventing the transmission of pathogens from food handlers to consumers. Food safety practices among food handlers were found to be generally low. Factors associated with poor food safety practices included sex, working environment, monthly income, regulatory supervision, food safety training, and attitude toward food safety^3^. A recent study has highlighted the prevalence of poor food handling practices among food handlers and significant associations between good food handling practices and factors such as marital status, food safety training, supervision by health professionals, routine medical checkups, and the level of knowledge of food handlers^4^. A study conducted in Bangladesh uncovered limited facilities, poor hygiene, and inadequate food-handling practices in restaurants and food vendor stalls^5^. Another study in Ethiopia found that the majority of food handlers did not adhere to proper food safety practices, including neglecting to check food temperatures and failing to wash their hands after sneezing^6^.

Research conducted in several countries indicated that most of the reported foodborne illness outbreaks originate from the types of food service establishments^7^. Therefore, food handlers have an important role in controlling these incidences. However, the control measures involve good food safety knowledge and a positive attitude towards hygiene practices during food handling and storage^8^. As per cognitive consistency theory, knowledge can be explained as what a person knows and how he feels and implements it^9^.

Due to a significant number of incidences of food-borne diseases, the safety of street food is a major concern for developing countries^10^. A study of street vended foods in Bangladesh found high microbial contamination in the collected samples, including strains resistant to multiple drugs.^11^.

Foodborne illnesses are often associated with inadequate food handling knowledge and expertise among food vendors. Some of the identified risk factors for foodborne illness include poor personal hygiene and sanitation, cross-contamination, inadequate cooking, improper storage, inadequate reheating, contaminated equipment, use of contaminated water, and purchasing raw foods from unreliable sources^12^. In most cases, street food vendors do not follow minimum hygiene practices, posing a high risk to the consumers’ health^13^. A previous investigation carried out in Barisal City, Bangladesh, highlighted the inadequate food safety practices observed among the street food vendors^14^.

Limited studies in Bangladesh focus on food safety practices and knowledge among street vendors and their impact on consumer health. Therefore, it is crucial to identify the factors that affect safe food handling practices, particularly during preparation and serving. The existing literature did not use an adequate number of variables to understand the scenario. In our study, we have incorporated several factors that could be linked to vendors' knowledge and practices, highlighting the distinctiveness of our research. Additionally, many previous studies were unable to comprehensively cover all areas of Dhaka city in a single article due to their sampling methods. By including ten busy spots in Dhaka city in our sampling methods, our study's scientific robustness is underscored. This study will add value to the existing evidence of food safety knowledge among street food sellers in Dhaka City.

## Method

### Study design

**Figure 1:**
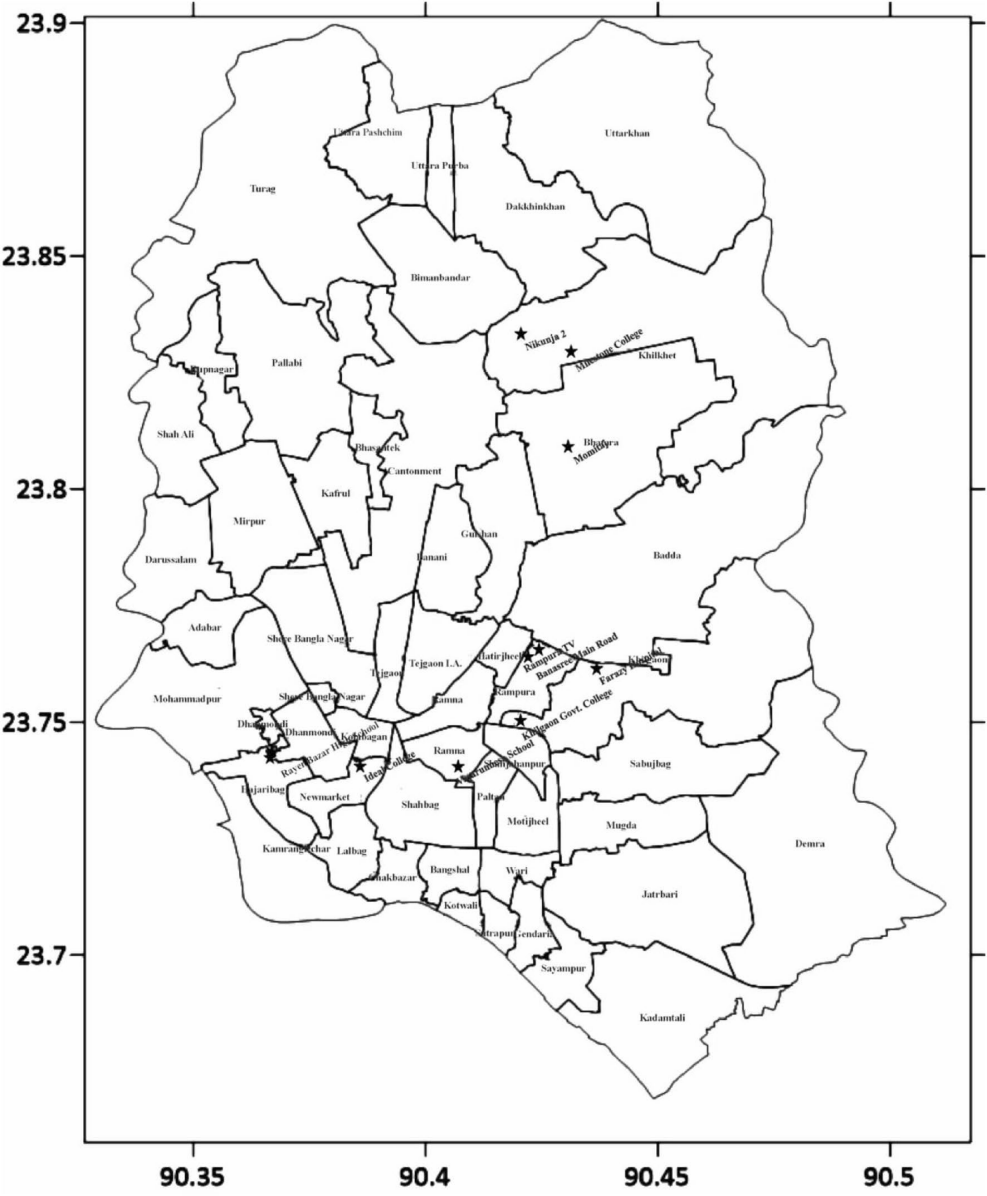
Study locations in Dhaka City, Bangladesh (marked as stars).

This cross-sectional study was conducted in seven locations in Dhaka City from February-September 2019. The study included the following locations in Dhaka: *Rampura, Banashree, Khilgaon, Siddeswari, Dhanmondi, Khilkhet and Basundhara* (Fig. 1).

### Ethical consideration

This study was part of a larger study conducted at the Department of Pharmacy, East West University. The ethics committee of East West University Center for Research and Training approved this study in January 2011 (CRT_Protocol_08_2011). The project followed the Helsinki Declaration of Ethical Principles, including participants’ autonomy, confidentiality of information, risk and benefits, and justice. The objectives and methods of data collection were fully described. An informed consent was obtained from all participants before enrollment.

### Research questions

The study addressed the following major research questions: (1) Do street food vendors know the importance of washing hands after handling raw materials, after cleaning food plates and tables, after handling garbage and before and after serving food? (2) Do street food vendors know the importance of wearing hand gloves? (3) Do food vendors know common health problems that can affect food safety? (5) What are their practices of hand washing and using hand gloves during food preparation, serving food, cleaning dishes, and handling garbage?

### Questionnaire

A questionnaire was developed and pretested for data collection. The external validity of the questionnaire was primarily determined by literature review, and the internal validity was based on feedback from relevant experts and researchers in the field. The questionnaire was pretested in a sample of 15 people from the same locality of the study, and necessary modifications were made.

The questionnaire was composed of the following major areas: (1) Demographics including gender, age, family composition, and major occupation; (2) Experience, education and training; (3) Food safety knowledge; (a) Importance of hand washing; (b) Importance of using hand gloves; (c) Knowledge about food-borne diseases; and (d) Knowledge about health problems that can affect food safety; (4) Practice of food handlers on food safety measures; and (5) An on-site observation of their practices.

Most of the questions were categorized as having yes, no, or don’t know answers. The survey questions consisted of 13 items to measure the knowledge scores on hand washing, 8 items for the use of hand gloves, 19 items for types of food-borne illnesses, and 10 items for types of health problems that can affect food safety. Non-probability convenient technique was used for the collection of data. The Cronbach’s Alpha was 0.74 on the questionnaire when considered separately in terms of knowledge and practice. A copy of the questionnaire is available in the appendix.

The survey was conducted by trained data collectors through in-person visits to the establishments. Three data collectors who had a minimum education of a bachelor’s degree interviewed the participants. The data collectors were trained in the use of the questionnaire, and they received guidance on how to establish rapport with respondents.

### Sample size estimation

For the baseline data, we used a cross-sectional study published by Nkosi and Tabit (2021) on the food safety knowledge of street food vendors in South Africa^15^. We used the conventional formula for cross-sectional study to calculate the sample size^16^, as follows:

$$n=\frac{{z}^{2}p (1-p)}{{d}^{2}}$$

where, n = sample size, z = Z statistic for a level of confidence, p = expected prevalence, and d = precision.

Based on the report, 76% of the street food vendors had low food safety knowledge; therefore, p = 0.76; 1—p = 0.24. Using 95% confidence, z = 1.96, and using a precision (or margin of error) of ± 5%, d = 0.05.

The calculated sample size = 281. To adjust for an anticipated 10% nonresponse rate, the total sample = 309. We used 350 samples for the study. The power of the study was 80% ^15,16^.

### Statistical analysis

All statistical analyses were performed using the Statistical Package for the Social Sciences, Version 28.0 (SPSS, Inc., Chicago, IL, USA). Data on knowledge were classified into several sections – (1) Importance of hand washing before and after different activities; (2) Importance of wearing hand gloves during different activities; (3) Knowledge of food-borne diseases; and (4) Type of health problems that can affect food safety. For each correct answer, the respondent scored one point and for an incorrect answer, the respondent received a zero point. A composite score of knowledge was calculated by adding the knowledge score of the four categories of knowledge scores. The scores were computed, and the relation between the knowledge scores were compared with the educational level of the participants, the qualification of the head chef, work experience, and the type of vending facility. Descriptive statistics were assessed for data distribution. The mean knowledge scores were compared by using one-way ANOVA. A post-hoc test (Tukey’s HSD) was computed for multiple comparisons and to identify which independent groups are significantly associated with the knowledge categories such as the practice of hand washing, wearing gloves, food-borne diseases, and type of health problems. Chi-square analysis was done to compare categorical variables such as each item of the four categories mentioned earlier and the head chef’s education status. A Pearson’s correlation was computed between the variables. Multiple regression analyses were done to predict the composite knowledge score based on education, work experience, and qualification of the head chef. Findings with a *p*-value of ≤ 0.05 were considered statistically significant.

## Results

### Demographic characteristics

The socio-economic and vending information (Table 1) of the participants show that most of the vendors were young adults aged 25 to 34 years. Regarding the educational level, 36.5% had completed primary education, and 47.7% had completed secondary education. Of the participants, 50.9% worked as chefs, 7.3% were cleaners, 23.3% were cutters, and 8.2% were food servers. Most participants (72.3%) said selling food on the street is their primary job. About half of them had 1 to 5 years of work experience, while 37% had 6 to 10 years of work experience. However, the majority (84.9%) never received food safety training.

**Table 1:** Socioeconomic and street vending background information of the study population.

Socioeconomic information	n (%)
Age (n = 330)	
< 18 years	3 (0.9)
19–24 years	50 (15.2)
25–29 years	127 (38.5)
30–34 years	136 (41.2)
35–39 years	14 (4.2)
Gender (n = 330)	
Male	324 (98.2)
Female	6 (1.8)
Education level (n = 329)	
No formal education	35 (10.6)
Primary	120 (36.5)
Secondary	157 (47.7)
Arabic Education (Madrasa)	17 (5.2)
Vending information	
Responsibility in food vending (n = 330)	
Chef	168 (50.9)
Washing utensils	24 (7.3)
Cutter	77 (23.3)
Server	27 (8.2)
Others	34 (10.3)
Main occupation (n = 318)	
Yes	230 (72.3)
No	88 (27.7)
Work experience (n = 330)	
< 1 year	42 (12.7)
1–5 years	166 (50.3)
6–10 years	122 (37.0)
Participation in food safety training/course (n = 324)	
Yes	49 (15.1)
No	275 (84.9)
Jobs done by family members (n = 330)	
Supervision	46 (13.9)
Cash holding	62 (18.8)
Purchases	135 (40.9)
Cooking	2 (0.6)
Serving	69 (20.9)
Cleaning	13 (3.9)
Not involved	3 (0.9)
Average no. of customers in a week (n = 329)	
21–99	43 (13.1)
100–199	165 (50.2)
200–299	52 (15.8)
300–399	57 (17.3)
400–499	12 (3.6)
No. of people associated with food handling (n = 297)	
1–2	100 (33.7)
2–3	125 (42.1)
3–4	57 (19.2)
> 4	15 (5.0)
Qualification of head chefs achieved through training (n = 325)	
Certified chef	56 (17.2)
Diploma or degree	8 (2.5)
Trained in restaurant	25 (7.7)
None	236 (72.6)
Type of vending facility (n = 328)	
Fixed stall	150 (45.7)
Mobile vending unit	169 (51.5)
Restaurant/canteen	9 (2.7)

### Food safety knowledge

**Figure 2:**
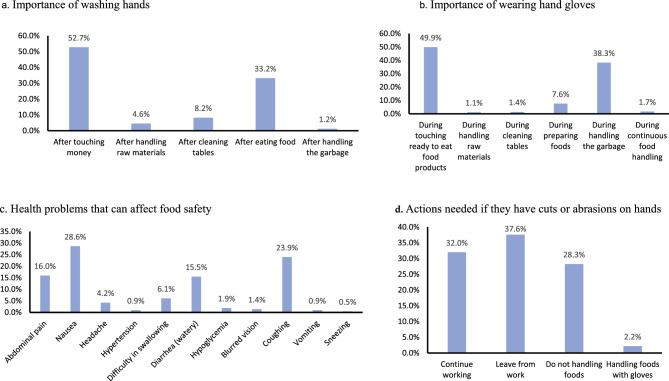
Food safety knowledge of the respondents. 2(**a**) Importance of washing hands, 2(**b**) Importance of washing gloves, 2(**c**) Health problems that can affect food safety, 2(**d**) Actions needed if they have cuts or abrasion on hands.

Figure 2 shows that about 53% of respondents mentioned that their hands should be washed after touching money, and 49.9% mentioned that they should wear gloves before touching ready-to-eat food products (Fig. 2). However, only a few mentioned the importance of washing hands after handling garbage (1.2%), raw foods (4.6%) and cleaning tables (8.2%). Health problems that can affect food safety were mentioned as follows: nausea (28.6%), coughing (23.9%), abdominal pain (16.0%), and diarrhoea (15.5%). Only 28% of the participants indicated that they should not handle foods, and a minority (2.2%) mentioned handling foods with gloves when they have cuts or abrasions on their hands, while 32% mentioned continuing work.

Table 2 shows that nearly 90% of the participants claimed to have experience of foodborne illnesses and knew the signs and symptoms of these diseases. The majority (76.8%) mentioned abdominal pain and 13.2% mentioned watery diarrhea as common symptoms of food-borne diseases. Although 45% said they would stop work immediately when they are sick, 18.2% of participants said they would continue to work.

**Table 2:** Knowledge and experience about food-borne diseases.

Variables	n (%)
Did you have experience with food-borne diseases (n = 328)	
Yes	291 (88.7)
No	37 (11.3)
Do you know the signs and symptoms of food-borne diseases (n = 327)	
Yes	292 (89.0)
No	37 (11.0)
What are the common signs and symptoms of food-borne diseases (n = 319)	
Abdominal pain	245 (76.8)
Nausea	18 (5.6)
Headache	7 (2.2)
Difficulty in swallowing	4 (1.3)
Diarrhea (watery)	42 (13.2)
Blurred vision	3 (0.9)
Complications that can happen due to food-borne diseases (n = 312)	
Respiratory failure	71 (33.5)
Kidney failure	93 (43.9)
Death	48 (22.6)
Do you continue working for food vending when you are sick (n = 330)	
Stop working on food handling immediately	149 (45.2)
Work throughout the sickness period	60 (18.2)
Work initially but stopped until symptoms disappeared	121 (36.7)
Can your health problems affect food safety (n = 319)	
Yes	199 (62.5)
No	119 (37.5)

### On-site observation of food handling practices

Table 3 shows that around 90% of the street vendors who were interviewed used gloves or washed their hands before handling food. However, about 77% only used gloves when handling ready-to-eat food products. About 5.6% of them were using gloves while handling raw foods. For cleaning cutting boards, 22.6% mentioned using detergents and hot water, and a large percentage (72.3%) mentioned rinsing the used knife under cold water only. Approximately 68% of the participants occasionally cleaned the spoons before using them again for the clients. Only 50.6% of the vendors used filtered water for cooking, compared to about 90.6% who used it for drinking. Approximately, 85% of them used tap water as the main source for cleaning utensils.

**Table 3:** Observed food handling practices of the vendors.

Variables	n (%)
Wash hands (n = 328)	
Yes	291 (88.7)
No	37 (11.3)
Wear gloves during food handling (n = 329)	
Yes	292 (89.0)
No	37 (11.0)
Wear gloves (n = 319)	
During touching ready to eat food products	245 (76.8)
During handling raw materials	18 (5.6)
During cleaning tables	7 (2.2)
During preparing foods	4 (1.3)
During cleaning utensils	42 (13.2)
During handling the garbage	3 (0.9)
Cleaning process of cutting board after cutting raw foods (n = 312)	
Wipe them on a tea towel/dishcloth	71 (33.5)
Rinse it under cold water	93 (43.9)
Wash it with detergent and hot water	48 (22.6)
Approaches to ensure that cutting boards used to cut raw foods are not subsequently used on foods that won’t be cooked (n = 330)	
Rinse it under cold water	149 (45.2)
Wash it with detergent and hot water	60 (18.2)
Wash it with detergent and hot water and mild bleach	121 (36.7)
Approaches to ensure that knives used to cut raw foods are not subsequently used on foods that won’t be cooked (n = 213)	
Two knives system	34 (16.0)
Wipe it on a tea towel / dishcloth	61 (28.6)
Wash after each use	9 (4.2)
Other	18 (0.5)
Dirty/used knife was cleaned (n = 328)	
Rinse it under cold water	237 (72.3)
Wash it with detergent and hot water	63 (19.2)
Wash it with detergent and hot water and mild bleach	10 (3.1)
Dishwasher or its equivalent	18 (5.5)
Clean your worktops by using (n = 295)	
Detergent	207 (70.1)
Washing up liquid	69 (23.4)
Sanitizer	19 (6.5)
Clean your hands after handling raw foods by (n = 325)	
Wipe them on a tea towel/dishcloth/j-cloth	120 (36.9)
Wash them with ordinary soap and hot/warm water	111 (34.2)
Wash them with antibacterial soap and hot/warm water	37 (11.4)
Do nothing	42 (12.9)
Other	15 (4.6)
Frequency of refreezing food after defrosting (n = 328)	
Once	137 (41.7)
Twice	30 (9.2)
Many times	7 (2.2)
Never	154 (46.9)
Duration of Food storage (n = 327)	
No storage at all	161 (49.2)
Half day to 1 weak	161 (49.2)
More than 1 weak	5 (1.6)
Spoons used in serving food to customers (n = 290)	
Used all day without washing	8 (3.3)
Washed occasionally and reused	199 (68.1)
Disposable spoon	83 (28.6)
Main sources of water used by you for cooking (n = 328)	
Tap water	161 (49.1)
Tanker/surface water (rivers, reservoirs and lakes etc.)	1 (0.3)
Filtered water	166 (50.6)
Main sources of water used by you for cleaning (n = 329)	
Tap water	279 (84.8)
Tanker/surface water (rivers, reservoirs and lakes etc.)	44 (13.4)
Filtered water	6 (1.8)
Main sources of water used by you for drinking (n = 326)	
Tap water	26 (8.1)
Tanker/surface water (rivers, reservoirs and lakes etc.)	4 (1.3)
Filtered water	296 (90.6)

### Factors associated with knowledge scores

Bivariate correlation analysis (Table 4) shows that the education level of the participants, work experience, and qualification of the head chef had a statistically significant relationship with knowledge about hand washing, wearing gloves, and food-borne illnesses (*p* < 0.001). However, no significant relationship has been achieved between work experience and knowledge about food-borne illnesses (*p* = 0.105).

**Table 4:** Bivariate correlation between variables of interest.

Variables	Work experience	Head chef’s qualification	Knowledge about		
			Hand washing	Wearing gloves	Foodborne illnesses
Education level	*r* = 0.177	*r* = -0.297	*r* = 0.223	*r* = 0.117	*r* = 0.212
	*p* = 0.001	*p* < 0.001	*p* < 0.001	*p* = 0.034	*p* < 0.001
Work experience		*r* = -0.291	*r* = 0.279	*r* = 0.220	*r* = -0.90
		*p* < 0.001	*p* < 0.001	*p* < 0.001	*p* = 0.105
Head chef’s qualification			*r* = -0.436	*r* = -0.406	*r* = -0.189
			*p* < 0.001	*p* < 0.001	*p* < 0.001
Hand washing				*r* = 0.861	*r* = 0.449
				*p* < 0.001	*p* < 0.001
Wearing gloves					*r* = 0.491
					*p* < 0.001

*r* = correlation coefficient.

### Overall knowledge score by categories of head chef’s qualification

Table 5 presents mean knowledge score calculated for four categories, such as knowledge on hand washing, knowledge on wearing hand gloves, knowledge on type of food-borne illness, and knowledge on health problems affecting food safety. Mean score of each of these categories were computed by using one-way ANOVA and Tukey’s HSD post hoc test. Overall, the knowledge score of handwashing was higher than the other areas of knowledge. The head chef’s qualifications, including having certified on food safety and being trained on job in the restaurant had significantly higher knowledge scores in each of the four categories compared with those without any such qualifications.

**Table 5:** Relation between food handler’s knowledge score and the head chef’s qualification.

	Mean (± SD) Knowledge Score by Categories of Head Chef’s Qualification			
Variable	None (a)	Certificate holder (b)	Trained in restaurant (c)	*p*- value*
Knowledge score on hand washing	6.05 ± 2.2	8.75 ± 0.7	9.6 ± 2.4	a vs. b, *p* < 0.001 a vs. c, *p* < 0.001
Knowledge score on wearing hand gloves	3.35 ± 2.56	6.29 ± 0.5	6.16 ± 2.9	a vs. b, *p* < 0.001 a vs. c, *p* < 0.001
Knowledge score on the type of food-borne illnesses	4.08 ± 1.8	5.28 ± 0.7	8.58 ± 5.7	a vs. b, *p* = 0.017 a vs. c, *p* < 0.001
Knowledge score on the type of health problems that can affect food safety	2.38 ± 2.8	3.59 ± 1.8	9.76 ± 6.5	a vs. b, *p* = 0.16 a vs. c, *p* < 0.001

*One-way ANOVA and Tukey’s HSD test.

### Multiple regression analysis to predict knowledge scores

A composite score of knowledge of the four categories, including knowledge on hand washing, wearing hand gloves, the type of food-borne illnesses and the type of health problems that can affect food safety, was used as the dependent variables, and the head chef’s education, work experience and qualification were used as independent variables in a multiple regression analysis, using a stepwise method of model selection. Education levels (no formal education, primary, secondary and madrasa education) (*p* = 0.014) and qualification (certificate, diploma, degree, on-job training, and none) (*p* < 0.001) were significant predictors of knowledge.

### Relation between individual items of the knowledge categories and the head chef’s qualification

For in-depth information, Chi-square analyses were conducted to find the relation between knowledge of individual items of each of the four categories and the head chef’s qualifications. Of the 15 questions (having yes and no answers) about knowledge of hand washing, 8 questions about wearing hand gloves, 19 questions about the types of food-borne diseases, and 20 questions on the types of health problems that can affect food safety, the head chef having certified on food safety provided significantly most correct answers on all items compared to chefs of other education categories, such as having a degree, diploma, trained on the job, or no education (p < 0.001 for all). A lack of knowledge of food handlers on many items related to food-borne diseases was identified. For example, they had adequate knowledge of common food-borne illnesses such as watery diarrhoea, nausea, and headache. In contrast, they had incorrect knowledge of items such as bloody diarrhoea, fatigue, and hypoglycemia, even when the head chefs were certified or diploma holders.

## Discussion

In this study, we observed that the head chef's education level, work experience, and qualification had a statistically significant relationship with the knowledge regarding hand washing, wearing gloves, and food-borne illnesses.

We also identified areas of inadequate knowledge and lack of healthy practices of street food vendors, which are likely to contribute toward the enormous burden of foodborne illnesses (such as watery diarrhoea, blood dysentery, salmonella infections, etc.)^5,17^.

Numerous studies have consistently identified food handlers as the main contributors to food-borne diseases by using improper food handling practices^2,3^. Our findings regarding the infrequent washing of utensils, supplies for preparing food, and vendor hygiene standards are consistent with earlier studies, suggesting the importance of the cleanliness of street food preparations in reducing the source of contamination^4^.

Formal education has been found to significantly impact the knowledge and adherence of street vendors to health safety measures aimed at preventing foodborne illnesses. The knowledge and practices of street vendors are crucial factors in ensuring food safety^18^. An earlier study has found a strong positive correlation between attitude and practice (r = 0.839, p < 0.01) and between knowledge and practice (r = 0.835, p < 0.01)^19^. Akabanda et al. showed a strong link between positive attitude, behaviour and education of food handlers in maintaining safe food handling practice^18^.

Our findings regarding the infrequent washing of utensils, supplies for preparing food, and vendor hygiene standards are consistent with earlier research suggesting that the uncleanliness of street food preparations may be the source of contamination^20^. Personal hand hygiene is the most important factor in preventing food contamination^21^. Most of the studies found a lack of hygiene practices in food handlers and consumers or mishandling of the foods during the preparation, storage, and vending^18,22^. The food hygiene methods of the vendors need to be prioritized and require ongoing assistance and regular inspections by the public health authorities. In addition, interventional studies that include school health programs and public educational campaigns are important to raise awareness about foodborne outbreaks and the significance of personal hygiene in preventing diseases^23^.

Our study was consistent with several studies from different countries that reported low knowledge scores of street food vendors. However, the data of the current study on the hygiene practice of food handlers cannot be directly compared with other studies due to the variations in research questions, study samples, and the primary objectives of the studies. For example, a study was conducted among food handlers in a catering company in Portugal^24^, responsible for manufacturing and distributing food to schools, kindergartens and nursing establishments. Contrary to our report, a study in Ghana by Akabanda and co-workers (2017)^18^ found that the food handlers demonstrated sufficient knowledge about hygiene practices such as hand washing, using gloves, and cleaning utensils properly. However, the sources for data collection of the Ghanaian population and our study differed. For example, the researchers in Ghana collected data on food handlers from various institutions such as schools, hospitals, universities, and health centers. On the other hand, we selected local street food vendors as our study participants. However, our study supports the findings of Nizame and his co-workers, who also demonstrated poor hygiene practices among street food vendors^5^. 

Approximately 30 million people in Bangladesh suffer from foodborne diseases each year^25^, and there is a lack of proper sanitary facilities at the vending sites in the country. A study from Vietnam underscores the value of providing training and education to street food sellers to enhance their knowledge of food safety and hygiene standards. Ensuring the availability of clean water, proper sanitation, and efficient waste disposal facilities is crucial for improving the infrastructure and services at street food vending sites^26^. In Bangladesh, the prevalence of foodborne diseases can be attributed to the lack of adequate sanitation and hygiene practices^27^.

Thus, it is essential to promote food safety practices in the production and consumption of street vended foods, especially in developing countries where hygiene standards are often in question^2^.

Unlike many previous studies, this research was carried out in seven areas of Dhaka City, which strengthens the validity of the data. Further studies are suggested to establish the link between specific pathogens found in street food samples and the illnesses experienced by consumers.

This study also provides data on safety concerns related to street vended food. In order to enhance the hygiene practices of food handlers in Bangladesh and other low-income countries, it is crucial to enforce the implementation of adequate toilet facilities with sufficient water supply and proper hand washing facilities. The regulatory authority must rigorously enforce effective training on food safety and the execution of hygiene practices. Food handlers need comprehensive health education to raise awareness about foodborne diseases, ensuring their health and that of consumers. They should receive appropriate training and keep all food-related items clean. The authority should designate proper food vending areas and provide proper sanitation and clean water near the vending areas. Designated garbage sites should be available for food vendors to dispose of their garbage properly. Food vendors should also always practice proper hygiene. By following these guidelines, food contamination can be reduced, customers will benefit, and food-related diseases will be less prevalent.

### Limitations

The present study had some limitations. As this is a cross-sectional study, we collected data after informing the study population about the purpose of our study, which might have resulted in reporting bias. Self-reporting of the vendors might be overestimated in their hygiene practices. Also, the study results should not be generalized to developed countries because of discrepancies in food hygiene regulations between developed and developing nations.

However, the study sample selection and data analysis methods were robust, as data from 10 spots in seven locations in busy city areas were included, and both univariate and multivariate analysis methods were employed. One of the unique aspects and novelty of this study is that we collected data through direct observation of food preparation and storage practices by the food handler.

## Conclusions

In this study, a composite score of knowledge related to hand washing, wearing hand gloves, types of food-borne illnesses, and health issues affecting food safety was significantly predicted by the level of education and qualifications of the food handlers. The study results were unique and novel in the context of Bangladesh, where the consumption of street food is quite common. This information can be used to develop intervention efforts in street foods and prevent foodborne disease. Findings from this study should be helpful for the policymakers to take initiatives for legal enforcement for the implementation of good hygiene practices of street vendors. The health authorities may initiate an ongoing and informative food vendors' training program. Food handlers must take the initiative to engage in self-learning and stay updated. Regulatory authorities responsible for food safety should conduct proper inspections and educate vendors about correct food safety practices. They should also look for specific inappropriate food handling practices identified in the study and assist the street food vendors in correcting them.

### Supplementary Information

Supplementary Information.

## Data Availability

The datasets used and analyzed during the current study are available from the corresponding author upon reasonable request.
