# Supplementary material for: Inadequate food safety knowledge and hygiene practices among street food vendors in Dhaka, Bangladesh
Source: Sci Rep. 2024 Jul 29;14:17349. doi: 10.1038/s41598-024-68099-y (PMC11284202; doi:10.1038/s41598-024-68099-y)
Supplement: Supplementary file 1 — Supplementary Information. [file 41598_2024_68099_MOESM1_ESM.docx]

**Appendix:**

**Street Vendors Survey Questionnaire**

**Basic occupational information….**

**1. Gender:** □ Male □ Female

**2. Educational level:** □ No formal education □ Primary □ Secondary □ Madhrasa

**3. Responsibility:** □ Chef □ Washing utensils □ Cutter □ Server □ Others

**4. Main occupation:** □ Yes □ No

**5. Work experience (years):** □ <1 □ 1-5 □ 6-10 □ >10

**6. Participation in food safety trainings/courses:** □ Yes □ No

**7. Your age groups**

| □ Under 18 | □ 19-24 | □ 25-34 |
| --- | --- | --- |
| □ 35-54 | □ 55-65 | □ Declined to answer |

**8. Jobs done by family members:**

□ Supervision & cash holding

□ Purchases □ Cooking

□ Serving □ Cleaning □ None

**9. What is the average number of customers who eat in the restaurant in typical weeks?**

□ Over 20 but less than 100 □ Over 100 but less than 200 □ Over 200 but less than 300 □ Over 300 but less than 400 □ Over 400 but less than 50 □ Over 500 but less than 1000

**10. How many people are associated in food handling operation in each restaurant?**

□ 1-2 □ 2-3 □ 3-4 □ More than 4

**11. Qualification that head chefs achieved through training:** □ Certificate □ Diploma □ Degree □ Trained in restaurant □ None

**12. Type of vending facility:** □ Fixed stall □ Mobile vending arrangement

□ Restaurant □ Canteen

**13. Which one is your priority?** □ Producing tasty food □ Producing safe food

**14. Do you think food safety knowledge can benefit your personal life?** □ Yes □No

**Food safety knowledge**

**15. It is important to wash hands**

| □ After touching money | □ After handling raw materials | □ After cleaning tables |
| --- | --- | --- |
| □ After eating food | □ After touching the body | □ After handling the garbage |
| □ Before preparing foods | □ During continuous food handling | □ After touching work clothes |
| □ After using toilets | □ After touching a clean countertop |  |
| □ Before using toilets | □ After blowing of nose |  |

**16. It is important to wear gloves**

| □ During touching ready to eat food products | □ During handling raw materials | □ During cleaning tables |
| --- | --- | --- |
| □ During preparing foods | □ During cleaning utensils | □ During handling the garbage |
| □ During continuous food handling | □ During touching work clothes |  |

**17. Sources of water used by food vendors should be**

| **For cooking** | **For cleaning/washing** | **For drinking** |
| --- | --- | --- |
| □ Tap water | □ Tap water | □ Tap water |
| □ Tanker/surface water (rivers, reservoirs and lakes etc.) | □ Tanker/surface water (rivers, reservoirs and lakes etc.) | □ Tanker/surface water (rivers, reservoirs and lakes etc.) |
| □ Filtered water | □ Filtered water | □ Filtered water |

**18. What should be the action of food handlers if they have abrasions or cuts on their hands?**

□ Continue working □ Take leave from work □ Do not handle foods □ Handle foods with gloves

**Knowledge of food-borne diseases**

**19. Do you have experience with food-borne diseases?** □ Yes □ No

**20. Do you know about the signs and symptoms of food-borne diseases?** □ Yes □ No

**21. Select the signs and symptoms of food-borne diseases:**

| □ Abdominal pain | □ Nausea | □ Headache | □ Hypertension | □ Difficulty in swallowing |
| --- | --- | --- | --- | --- |
| □ Diarrhea (watery) | □ Hypoglycemia | □ Coughing or sneezing | □ Blurred vision | □ Vomiting |
| □ Diarrhea (bloody) | □ Pain in the bone | □ Muscle weakness | □ Loss of appetite | □ Loss of weight |
| □ Fatigue | □ Bleeding within the skin | □ Muscle aches | □ Fever |  |

**22. Do you think food-borne diseases can lead to:**

□ Respiratory failure □ Kidney failure □ Death

**23. Did you stop working during illness?**

□ Stopped working on food handling immediately

□ Didn’t stop working; continued working throughout the sickness period

□ Worked initially but stopped until symptoms disappeared

**24. Do you think your health problems can affect food safety?** □ Yes □ No

**25. Which of the following health problems can affect food safety?**

| □ Abdominal pain | □ Nausea | □ Headache | □ Hypertension | □ Difficulty in swallowing |
| --- | --- | --- | --- | --- |
| □ Diarrhea (watery) | □ Hypoglycemia (Low blood sugar) | □ Coughing | □ Blurred vision | □ Vomiting |
| □ Diarrhea (bloody) | □ Pain in the bone | □ Muscle weakness/pain | □ Wound covered with bandage | □ Sneezing |
| □ Smoking | □ Fever | □ Open wound | □ Covered wound in the hand with wearing a glove | □ Sore throat |

**Practice**

**26. When should you wash your hands?**

| □ After touching money | □ After handling raw materials | □ After cleaning tables |
| --- | --- | --- |
| □ After eating food | □ After touching the body | □ After handling the garbage |
| □ Before preparing foods | □ During continuous food handling | □ After touching work clothes |
| □ After using toilets | □ After touching a clean countertop |  |
| □ Before using toilets | □ After blowing of nose |  |

**27. Do you wear gloves during food handling?** □ Yes □No

**28. When do you wear gloves?**

| □ During touching ready to eat food products | □ During handling raw materials | □ During cleaning tables |
| --- | --- | --- |
| □ During preparing foods | □ During cleaning utensils | □ During handling the garbage |
| □ During continuous food handling | □ During touching work clothes |  |

**29. After cutting raw foods, what do you do with your cutting board?**

□ Wipe it with a tea towel/dishcloth

□ Rinse it under cold water

□ Wash it with detergent and hot water

□ Wash it with detergent and hot water and mild bleach (8%)

□ Do nothing

**30. How do you ensure that the cutting boards after cutting raw foods are not subsequently used on foods that won’t be cooked?**

□ Rinse it under cold water

□ Wash it with detergent and hot water

□ Wash it with detergent and hot water and mild bleach 8%

□ Different cutting boards are used for different purposes (Color coded)

**31. What materials are your cutting boards made of?** □ Plastic □ Wood □ Glass

**32. How do you ensure knives used to cut raw foods are not subsequently used on foods that won’t be cooked?**

□ Use two knives system □ Wipe it with a tea towel/dishcloth □ Wash after each use

□ Other, Specify _________________

**33. How do you clean the dirty/used knife?**

□ Rinse it under cold water

□ Wash it with detergent and hot water

□ Wash it with detergent and hot water and mild bleach

□ Use dishwasher or its equivalent

**34. How do you clean your worktops?** □ Use detergent □ Use only liquid □ Sanitize

**35. How do you clean your hands after handling raw foods?**

□ Wipe them with tea towel/dishcloth/j-cloth

□ Wash them with ordinary soap and hot/warm water

□ Wash them with antibacterial soap and hot/warm water

□ Do nothing

□ Other

**36. How many times do you refroze food after defrosting?**

□ Once □ Twice □ Many times □ Never

**37. How long do you store food?**

□ No storage at all □ Half day to 1 week □ More than 1 week

**38. Spoons used in serving food to customers:**

□ Used all day without washing □ Washed occasionally and reused □ Used disposable spoon

**39. Main sources of water that you use:**

| **For cooking** | **For cleaning/washing** | **For cooking/drinking** |
| --- | --- | --- |
| □ Tap water | □ Tap water | □ Tap water |
| □ Tanker/surface water (rivers, reservoirs and lakes etc.) | □ Tanker/surface water (rivers, reservoirs and lakes etc.) | □ Tanker/surface water (rivers, reservoirs and lakes etc.) |
| □ Filtered water | □ Filtered water | □ Filtered water |

**40. What do you do if the food handlers have abrasions or cuts on their hands?**

□ Continue working □ Take leave from work □ Don’t handle foods □ Handle foods with gloves

**41. How can you ensure food safety/ prevent food-borne disease?**

| □ Obtain more food safety knowledge | □ Washing hand before handling raw or cooked foods | □ Use different clean clothes to mop dining tables and food utensils |
| --- | --- | --- |
| □ Attend training regarding food hygiene/safety | □ Storing foods properly | □ Attend training regarding sanitation |
| □ Practicing good personal hygiene | □ Keeping raw and cooked foods separately | □ Washing hands before work |
| □ Taking leave when sick, fever or catch cold | □ Know the temperature chillers and freezers | □ Sanitize knives and cutting boards properly |
| □ Using caps, masks, protective gloves and adequate clothing | □ Check temperature settings of chillers and freezers regularly | □ Washing utensils with detergent |
| □ Changing incorrect food handling practices |  |  |
